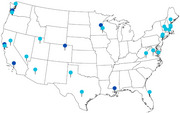# Facilitators and challenges to implementing and scaling collaborative dementia care in diverse health systems: Lessons from the Care Ecosystem

**DOI:** 10.1002/alz70858_102202

**Published:** 2025-12-25

**Authors:** Katherine L. Possin, Sarah Dulaney, Michelle Barclay, Alma V Hernandez De Jesus, Alissa Bernstein Sideman

**Affiliations:** ^1^ Memory and Aging Center, Weill Institute for Neurosciences, University of California, San Francisco, San Francisco, CA, USA; ^2^ Global Brain Health Institute (GBHI), University of California San Francisco (UCSF); & Trinity College Dublin, San Francisco, CA, USA; ^3^ University of California San Francisco, San Francisco, CA, USA; ^4^ University of California, San Francisco, San Francisco, CA, USA

## Abstract

Collaborative care models offer an evidence‐based solution for health systems to address the complex medical and social needs for people living with dementia (PLWD) and their caregivers. Widespread implementation is a challenge. The Care Ecosystem team has taken a unique approach by providing open‐source implementation materials and monthly implementation support meetings to program leads since 2018. Applying an anthropological approach, we evaluated the facilitators, challenges, adaptations, and sustainability plans when implementing the Care Ecosystem model across 28 health systems via observation of the monthly meetings, review of meeting minutes, and in‐depth interviews with the leads and team members at six of the health systems (*n* = 16). Health system locations are shown in the figure, with dark blue pins for programs that participated in the interviews. Facilitators to implementation included having a clinical champion advocating for practice change; a team that included care navigators with an aptitude for rapport‐building, a member with strong project management skills, and dementia clinicians; the open‐source implementation materials, including care protocols and online training; the flexibility of the Care Ecosystem model; and the collaborative and supportive learning environment provided by the monthly meetings. Challenges included the time and resources required for program start‐up, staff turnover and burn out, and financial sustainability. Adaptations to the care model were common and included alternative staffing models, shorter or less intense intervention dosage, and tailoring of protocols and caregiver education to serve specific populations. To achieve sustainability, programs collected and analyzed data on metrics important to their health systems to form the business case. Programs also applied new billing codes, supplemented funding with philanthropy and grants, and 20 of the programs were selected as participants in Medicare's new GUIDE payment model. Sites that chose not to participate or withdrew after being selected from GUIDE cited challenges in setting up respite contracts and in‐home service requirements and concerns that GUIDE payments would not cover program costs. Insights from this work can help clinical innovators identify ways to strengthen and support their teams, adapt their models to their local context, and begin to address sustainability issues early on.